# Impact of Device Architecture on Proton Detection Efficiency in 2D Perovskite Thick Film Detectors

**DOI:** 10.1002/smll.202512236

**Published:** 2026-01-25

**Authors:** Giulia Napolitano, Sara Cepić, Ilaria Fratelli, Massimo Chiari, Beatrice Fraboni, Laura Basiricò

**Affiliations:** ^1^ Department of Physics and Astronomy University of Bologna Italy; ^2^ INFN Sezione di Bologna Bologna Italy; ^3^ INFN‐Florence Sesto Fiorentino Florence Italy

**Keywords:** 2D Perovskites, stacked architectures, thin films, detectors, protons

## Abstract

Hybrid organic–inorganic perovskites have emerged as promising materials for ionizing radiation detection due to their excellent optoelectronic properties, ease of performance tunability, and fabrication onto flexible substrates. In this study, we compare two device architectures, planar and stacked, for the direct detection of 5 MeV protons using thin films of 2D perovskite PEA_2_PbBr_4_ (PEA = C_6_H_5_C_2_H_4_NH_3_^+^). We demonstrate that the stacked configuration, with a vertical electric field across the perovskite layer, enables superior charge collection and significantly enhances detection performance compared to the lateral planar geometry. Proton irradiation experiments conducted over a wide range of fluxes (10^8^–10^1^ ^0^ H^+^ cm^−^^2^ s^−^^1^) confirm the improved sensitivity, reproducibility, and long‐term operational stability of the stacked devices, particularly for thinner active films where morphological uniformity is higher. Additionally, stacked detectors exhibit a stable and energy‐independent response across the tested energy range (3, 4, and 5 MeV), indicating efficient charge transport and collection mechanisms, irrespective of the proton linear energy transfer. These results emphasize the key role of device geometry and highlight how the stacked configuration is a robust and scalable solution for next‐generation proton dosimeters.

## Graphical abstract

This work shows the role of device architecture in MeV proton detection with 2D perovskite thick (> 1 µm) films. A direct comparison between planar and stacked devices demonstrates that the stacked geometry achieves superior response by enabling more efficient charge collection and better exploitation of the active layer volume, ensuring stable and reproducible performance.

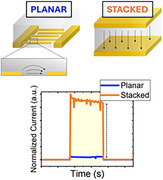

## 1 Introduction

The detection of low‐energy protons is crucial in personal dosimetry: for example, these particles constitute a major component of secondary radiation in space environments, define the end‐of‐range in proton therapy, and arise abundantly from neutron interactions in accelerator facilities [1, 2, 3]. These are all scenarios that often benefit from detectors that can conform to curved or extended surfaces [4, 5, 6, 7, 8, 9, 10]. However, conventional inorganic semiconductor detectors are rigid and expensive, limiting their use in flexible or large‐area dosimetry [11]. In this context, solution‐processed hybrid perovskites offer low‐cost fabrication [12, 13], scalability [14, 15, 16, 17] and mechanical flexibility [18, 19, 20, 21, 22, 23], and 2D perovskites (with structural formula (R‐NH_3_)_2_A_n‐1_B_n_X_3n+1_, where R is an alkyl or aryl group) additionally provide enhanced stability, low dark current and suppressed ion migration, making them particularly promising for these applications [24, 25, 26, 27, 28]. Indeed, recent studies have successfully demonstrated their potential for the direct detection of X‐rays, γ‐rays, fast neutrons, protons, and other charged particles and the realization of real‐time dosimeters [29, 30, 31, 32, 33, 34, 35].

A key aspect in optimizing perovskite‐based proton detectors is increasing the thickness to maximize the interaction between the incoming radiation and the active material. However, a thicker absorber also requires a geometry device capable of efficiently collecting charges generated throughout the full film depth. Most perovskite polycrystalline film detectors rely on co‐planar electrodes, where the electric field lies parallel to the film surface [36, 37]. This layout collects carriers mainly near the electrodes and becomes increasingly inefficient as the active layer becomes thicker [38, 39]. A stacked architecture overcomes these limitations by applying the electric field perpendicularly to the film, enabling full‐volume charge collection and making the use of thicker layers an effective strategy to increase the detecting efficiency. The benefits of stacked layouts have already been demonstrated in several materials and device architecture [40, 41, 42, 43]. Specifically, for what concerns ultrathin perovskite films, Yakunin et al. [44] first showed their effectiveness for X‐ray detection, and subsequent works by Demchyshyn et al. [45] and Verdi et al. [46] confirmed their excellent sensitivity and the possibility to employ them under low‐bias operation. However, all these examples rely on films thinner than 1 µm, where carrier transport distances remain short and thickness‐dependent losses are minimal.

For solution‐processed 2D perovskite films, which exhibit more isotropic transport than single crystals [47, 48], extending stacked geometries to significantly thicker layers would be highly advantageous but remains largely unexplored. Indeed, achieving uniform thick films, preventing short circuits, and maintaining efficient out‐of‐plane transport present non‐trivial challenges.

In this work, we present a comparative study of the impact of device geometry on the detection performance of 2D perovskites. We design, fabricate, and compare two perovskite‐based detector architectures, planar and stacked, tested under 5 MeV proton irradiation. The active layer in both configurations consists of a 2D layered perovskite, PEA_2_PbBr_4_ (PEA = C_6_H_5_C_2_H_4_NH_3_^+^), polycrystalline film [49, 50]. We recently demonstrated that this material can be effectively employed for the direct detection of protons and real‐time beam monitoring, highlighting its suitability for integration into compact and low‐cost dosimetry systems [33, 51]. This work aims to show how, through an accurate design and control, the stacked geometry can enhance charge collection by fully exploiting the active material volume and ensure a uniform electric field across the semiconductor thickness. Such a comparison between the two architectures was made possible thanks to the excellent control achieved over the film deposition process, which ensured uniform thickness and high‐quality morphology across thicker layers (up to 10 µm), up to two orders of magnitude higher than those used in previous studies on stacked architectures. Our results reveal that the stacked architecture significantly outperforms the planar configuration in terms of proton‐induced signal intensity and stronger electric field‐driven improvement in charge collection. Therefore, they show that these concept can be reliably implemented in solution‐processed 2D perovskite thick‐film devices operating under MeV proton irradiation, a combination that had not been previously explored. Stacked detectors exhibit excellent monitoring of the proton beam, reproducibility, and long‐term operational stability. Overall, this study demonstrates that the implementation of the most suitable device architecture represents a key strategy for boosting the performance of perovskite‐based detectors and that the stacked layout offers a promising route for the development of efficient proton sensors.

## 2 Results and Discussion

**FIGURE 1:**
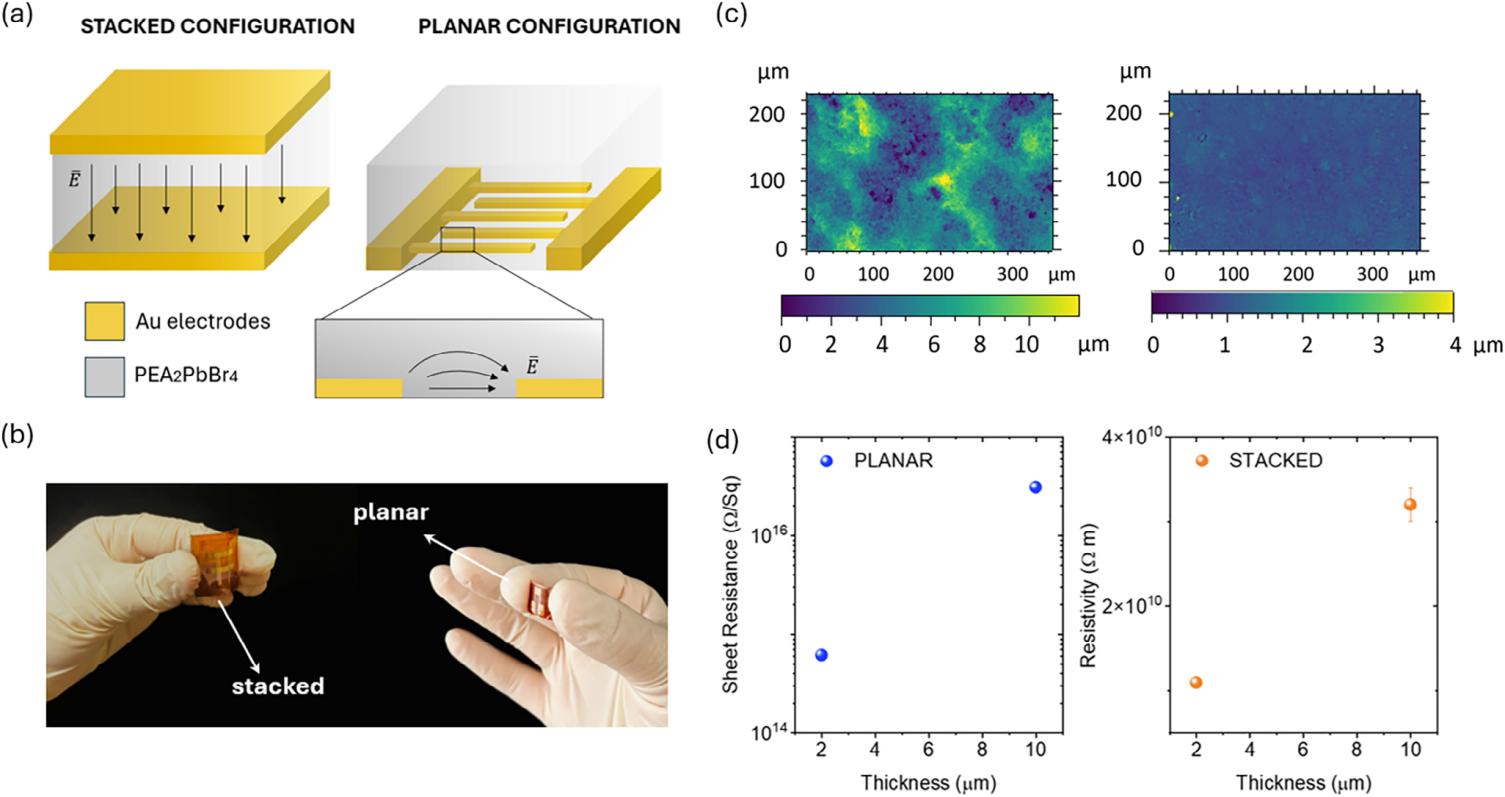
(a) Schematic representation of two different detector architectures for a thin film 2D hybrid perovskite ((PEA)_2_PbBr_4_) photoconductor. On the right, an interdigitated Au electrode planar structure (L = 30 µm, W = 50 mm, pixel area = 2 × 2 mm^2^), where the electric field is applied laterally across the photoconductive material; on the left, a stacked Au/(PEA)_2_PbBr_4_/Au structure, where the electric field is applied along the thickness of the semiconductor layer. (b) Image showing the two devices. (c) Top surface morphology for 10 µm (left) and 2 µm (right) perovskite films, acquired by an optical profilometer. (d) Plot of the sheet resistance and resistivity values, respectively for planar (blue) and stacked (orange) architectures, both for 2 and 10 µm perovskite thin films.

The planar and stacked detector configurations under study, along with their respective schematic of electric field line patterns, are presented in Figure 1A. Both employ a 2D hybrid perovskite (PEA_2_PbBr_4_) active layer. The electrodes were fabricated by depositing Cr/Au via thermal evaporation onto 75 µm thick polyimide substrates (KAPTON). Specifically, in the planar layout, interdigitated electrodes were patterned through photolithography (L = 30 µm, W = 50 mm, pixel area = 2 × 2 mm^2^), followed by spin‐coating of the perovskite polycrystalline film directly on top. For the stacked configuration, a bottom Cr/Au electrode was thermally evaporated through a shadow mask. The perovskite layer was then deposited by spin coating using the same procedure as in the planar device. Finally, the top Au electrode was thermally evaporated through a shadow mask of identical area to define the active region and complete the vertical architecture. The energy‐level alignment at the Au/PEA_2_PbBr_4_ interface was considered according to values recently reported for PEA_2_PbBr_4_ single crystal [52], which provide experimentally measured valence and conduction band positions for this material. In particular, they determined a valence band maximum (VBM) of –6.30 eV, a conduction band minimum (CBM) of –3.45 eV, and an optical bandgap of ≈ 2.9 eV for PEA_2_PbBr_4_, together with an Au work function of –5.1 eV. The pictures of the final devices are shown in Figure 1B. Samples with different thicknesses were fabricated by tuning the speed of the spin coating deposition. In particular, the active layers were deposited at 800 and 6000 rpm spin velocity, to vary the thickness from 10 to 2 µm, respectively. Thickness measurements were performed using an optical profilometer: for the lowest spin velocity (800 rpm), the thickness is (10.3 ± 0.2) µm for the planar configuration and (10.4 ± 0.1) µm for the vertical one. At 6000 rpm, the thickness decreases to (2.1 ± 0.1) µm for the planar samples and (1.99 ± 0.02) µm for the vertical samples (Figure S1). Additional details about the fabrication process are included in the Experimental Methods section. Profilometric analysis also allowed the evaluation of surface roughness (Figure 1C), revealing a difference in morphology between the samples with different thicknesses. The stacked thickest film (top left) shows a higher surface roughness (2.8 ± 0.5 µm) and reduced uniformity compared to the thinner one (top right) (0.25 ± 0.02 µm), which displays a more compact and continuous structure. Same values were observed for the planar configuration, with roughness values of 2.9 ± 0.2 and 0.25 ± 0.06 µm for the 10 and 2 µm films, respectively. Additional optical profilometry maps acquired at two regions located at opposite edges of the 2 cm^2^ substrate are provided in Figure S2, confirming that the films show comparable morphology across the full substrate area.

AFM measurements were also performed to further assess the surface morphology (Figure S3), confirming the thickness‐dependent evolution of the morphology, showing smoother and more homogeneous surfaces in the 2 µm films and a more granular texture in the 10 µm layers, consistent with the roughness extracted by optical profilometry.

The crystallinity and phase purity of the deposited PEA_2_PbBr_4_ films were verified by X‐ray diffraction (XRD) for both 10 and 2 µm. Figure S4 shows the XRD patterns of the films measured under ambient conditions. All samples display the reflections characteristic of the layered PEA_2_PbBr_4_ phase, with the main peaks located at 2θ = 10.64°, 15.88°, 21.25°, 26.73°, 32.23° and 37.70° [53], corresponding to the (001)–(007) planes, respectively. Additional reflections originate from the Kapton substrate, appearing at 2θ = 14.01°, 16.83°, 18.58°, and 25.53° [54]. A slight amorphous halo is visible as a broad elevation of the baseline at low angles; this feature is commonly associated with residual amorphous organic components—most plausibly trace amounts of solvent remaining in the film [55, 56, 57]. These measurements confirm that the films adopt the expected 2D Ruddlesden–Popper structure while retaining the morphology typical of solution‐processed polycrystalline PEA_2_PbBr_4_.

We also performed SEM analysis to acquire top‐view and cross‐section images of the two samples (Figure S5). In addition to confirm the film thickness extracted from optical profilometry, the cross‐sectional SEM shows how the polycrystalline PEA_2_PbBr_4_ films used in this work do not exhibit perfectly parallel 2D layers, but rather a distribution of slightly tilted crystallites. This tilted‐grain morphology is a well‐known feature of solution‐processed 2D perovskite films (e.g. Chen et al. [58]) and plays a crucial role in enabling out‐of‐plane transport. Unlike single‐crystal 2D perovskites [47, 48], where the inorganic slabs lie strictly in‐plane and vertical mobility is strongly suppressed, polycrystalline films provide a network of misaligned slabs that allows vertical percolation pathways. The orientation of these microstructures explains why film devices, rather than single crystals, are suitable for stacked architectures.

To investigate the transport properties of the two architectures, electrical characterization was performed under dark conditions. In planar devices, where the current flows laterally along the film, the sheet resistance (*R_S_*) was extracted from the current‐voltage (IV) curves, using the relation $\left(R_{S}=R\frac{W}{L}\;\right)$, where R is the resistance and L and W are respectively the channel length and width. This parameter is particularly relevant in lateral geometries, as it describes the resistance per unit square of material and reflects the in‐plane transport characteristics of the active layer. In contrast, in the stacked configuration, the current flows perpendicularly through the film thickness, allowing for the direct extraction of the bulk resistivity (*ρ*) of the material. Specifically, the sheet resistance extracted is (3.1 ± 0.4) × 10^16^ Ω Sq^−1^ for the 10 µm thick and (6.2 ± 0.1) × 10^14^ Ω Sq^−1^ for the 2 µm thick planar configurations, while the resistivity is (3.2 ± 0.2) × 10^10^ Ω m for 10 µm and (1.11 ± 0.06) × 10^10^ Ω m for the 2 µm stacked structures. As shown in Figure 1D, thinner films exhibit lower sheet resistance and resistivity in the stacked and planar configurations, respectively, which can be attributed to their improved morphological uniformity shown in Figure 1C. The mobility–lifetime product measured for analogous devices, with a 2 µm‐thick PEA_2_PbBr_4_ active layer, is on the order of 10^−^^5^ cm^2^ V^−^^1^, as previously reported in our earlier work [49]. This value is fully consistent with those typically observed in benchmark materials used for large‐area radiation detection, such as amorphous selenium (α‐Se) [59].

**FIGURE 2:**
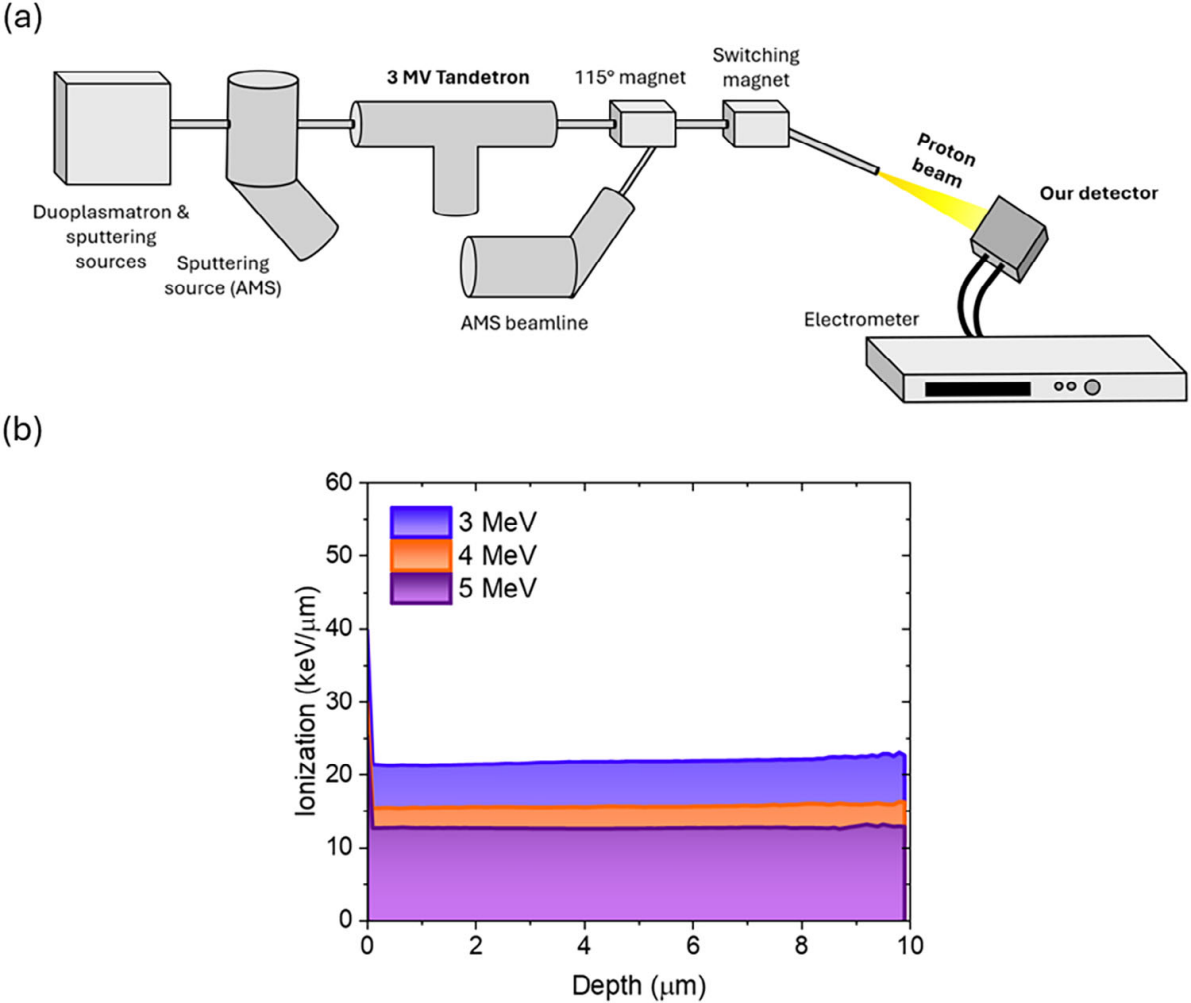
(a) Schematic representation of the 3 MV Tandetron accelerator beamline at LABEC (INFN, Florence), used to irradiate samples with 3–5 MeV protons. The proton beam is focused and directed onto the detector under test, enclosed in a Faraday cage, and the current is monitored by an electrometer. (b) Ionization profiles simulated using SRIM, showing the energy loss per unit length (keV µm^−1^) as a function of depth for proton beams of 3 MeV (blue), 4 MeV (orange), and 5 MeV (purple).

The response under protons was characterized at LABEC ion beam center (Laboratory of Nuclear Techniques for the Environment and Cultural Heritage, INFN Firenze, Italy) [60] using the 3–5 MeV proton beam provided by the 3 MV Tandetron accelerator. This beam energy was selected because low‐energy protons have a high linear energy transfer (LET) in matter, leading to greater energy deposition along their path. The increased ionization density results in a higher number of charge carriers generated per unit length within the detector material. Such high absorption conditions allow a reliable evaluation of differences in charge collection efficiency, mobility, and recombination dynamics between the devices under study, making this facility particularly suitable to compare different detector architectures. According to the Bethe‐Bloch [61] formula, the LET is approximately inversely proportional to the square of the proton velocity,

$$-\left(\frac{dE}{dx}\right)=\frac{4\pi N_{A}z^{2}}{m_{e}c^{2}\beta^{2}}\frac{Z\rho }{M_{u}A}{\left(\frac{e^{2}}{4\pi \varepsilon_{0}}\right)}^{2}\left[ln\left(\frac{2m_{e}c^{2}\beta^{2}}{I\left(1-\beta^{2}\right)}\right)-\beta^{2}\right]$$

where $\beta =\frac{v}{c}\;$ is the reduced particle velocity. As the proton energy decreases, *β* decreases, resulting in a higher LET. A schematic of the experimental setup is provided in Figure 2A. During the measurements, the detectors were placed inside a metal box, acting as a Faraday cage to screen the electrical noise and keep them in the dark. The sensor was aligned with the proton beam axis to ensure uniform exposure. The actual energy of the protons reaching the active layer of the detector was estimated from the calculation of the energy lost by each proton through several interposed media. Based on Stopping and Range of Ions in Matter (SRIM) [62] Monte Carlo simulations, the total energy loss by 5 MeV protons through these layers amounts to approximately 390 keV. The LET released by each 5, 4, and 3 MeV proton inside the 10 and 2 µm perovskite layers was simulated by SRIM as well. The results are shown in Figure 2B and summarized in Table 1. Specifically, for the stacked architecture we included in the simulation a 40 nm thick gold layer to account for the top electrode; however, its effect is considered negligible, as no significant differences were observed in the energy released because of its presence (Figure S6).

**TABLE 1:** Table showing the integrated ionization energies at selected depths (2 µm and 10 µm), for each proton energy.

Proton Beam Energy (MeV)	Ionization (keV) @10 µm	Ionization (keV) @2 µm
5	125	25
4	153	30.6
3	211	42.2

### 2.1 Comparison between Planar and Stacked Architecture

**FIGURE 3:**
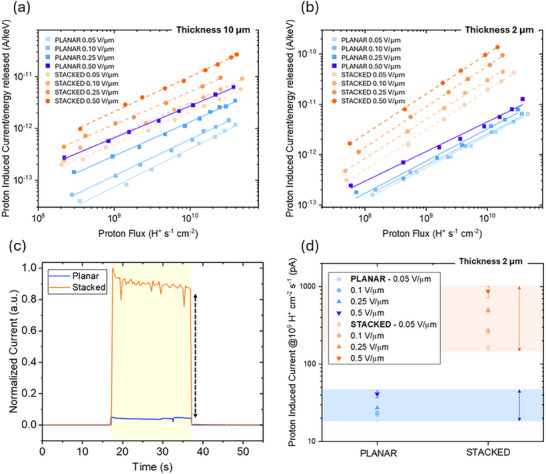
Plot of 5 MeV proton‐induced current, normalized by the energy released in the active layer, as a function of flux for detectors with a 10 µm (a) and 2 µm (b) thick perovskite layer. The data compares planar and vertical configurations under different nominal applied electric fields (defined as the applied voltage divided by the channel length), ranging from 0.05 to 0.5 V µm^−1^. (c) Comparison between the current vs. time response of the detectors in the planar (blue) and stacked (orange) configurations, normalized to the maximum of the proton induced current. The data refer to the 2 µm thickness, under a nominal electric field of 0.5 V µm^−1^ and a proton flux of 10^10^ H^+^ s^−1^ cm^−2^. The yellow region corresponds to the irradiation window. (d) Plot showing the proton‐induced current values obtained for the 2 µm thick perovskite layer in planar (blue) and stacked (orange) configurations, at 3 ×10^9^ H^+^ cm^−2^ s^−1^ under different nominal applied electric fields, ranging from 0.05 to 0.5 V µm^−1^. Shaded areas indicate the range of the signal variation; arrows highlight the stronger electric field dependence in stacked devices.

To directly compare the performance of the planar and stacked architectures, we tested both configurations under a 5 MeV proton beam, varying the nominal electric field, calculated as the applied bias divided by the channel length (from 0.05 to 0.5 V µm^−1^) and the incident proton flux (ranging from 10^8^ to 10^1^ ^0^ H^+^ cm^−^^2^ s^−^^1^). For both 2 µm and 10 µm thick devices, we observed a consistently higher response in the stacked configuration. This trend clearly emerges from the curves of proton‐induced current vs. proton flux shown in Figure 3A,B, which are normalized to the energy released in the active layer. Specifically, in the 2 µm thick structures, the stacked architecture shows a significantly higher response across all fluxes and fields, demonstrating its superior ability to collect charges generated throughout the volume. This advantage becomes less pronounced in the 10 µm devices, where both configurations suffer more from incomplete collection. As shown in Figure S7, the vertical device exhibits a higher normalized response when the thickness is reduced from 10 to 2 µm, across all applied electric fields. This behavior can be attributed to less efficient charge transport in the thickest layer. The worse transport in 10 µm devices is likely related to morphological limitations, as confirmed by the profilometer images of the thin film perovskite surface (Figure 1C). Such morphological disorder may affect the charge collection efficiency as demonstrated by the higher resistivity of the thicker film reported in Figure 1D, thus explaining the observed performance drop. Nevertheless, for the same thickness, the stacked configuration performs better than the planar one.

The detector sensitivity, calculated as the maximum of the first derivative of the proton induced current (*I*) vs. proton flux (*Φ*) $(S=\frac{1}{A}\;\frac{dI}{d\Phi })\;$ at 0.5 V µm^−1^, results (1.0 ± 0.2) × 10^−17^ C H^+^^−1^ for 2 µm thick stacked configuration and (7.19 ± 0.05) × 10^−19^ C H^+^^−1^ for the planar one. Instead, for the 10 µm thick film, the sensitivity value is (5.7 ± 0.1) × 10^−18^ C H^+^^−1^ for stacked and (1.52 ± 0.03) × 10^−18^ C H^+^^−1^ for planar detectors. The metric C H^+‐1^ refers to the charge collected per incident proton. The raw current vs. flux data used to extract the sensitivity are provided in Figure S8. The sensitivity values are reported in Table 2 along with thickness, surface roughness, resistivity, and sheet resistance.

**TABLE 2:** Summary of thickness, surface roughness, electrical resistivity and sheet resistance, and proton sensitivity for both stacked and planar device architectures. The sensitivity was reported for 5 MeV proton beam energy and 0.5 V µm^−1^ electric field applied.

STACKED				
	Thickness (µm)	Roughness (µm)	Resistivity (Ω m)	Sensitivity (C H^+^^−1^)
STACKED 2 µm	1.99 ± 0.02	0.25 ± 0.02	(1.11 ± 0.06) × 10^10^	(1.0 ± 0.2) × 10^−17^
STACKED 10 µm	10.3 ± 0.2	2.8 ± 0.5	(3.2 ± 0.2) × 10^10^	(5.7 ± 0.1) × 10^−18^
**PLANAR**				
	**Thickness (µm)**	**Roughness (µm)**	**Sheet Resistance (Ω Sq^−1^)**	**Sensitivity (C H^+^^−1^)**
PLANAR 2 µm	2.1 ± 0.1	0.25 ± 0.06	(3.1 ± 0.4) × 10^16^	(7.19 ± 0.05) × 10^−19^
PLANAR 10 µm	10.4 ± 0.1	2.9 ± 0.2	(6.2 ± 0.1) × 10^14^	(1.52 ± 0.03) × 10^−18^

This sensitivity value is higher than the one achieved by TIPGe‐pentacene planar proton detector, measured under an electric field of 0.03 V µm^−1^ (S = (6.4 ± 0.2) × 10^−20^ C H^+^^−1^) [63]. Furthermore, it is comparable to the sensitivity achieved by a vertical detector based on MAPbBr_3_ thick single crystal irradiated using 3 MeV protons and biased at 0.01 V µm^−1^ (S = (2.19 ± 0.03) × 10^−18^ C H^+^^−1^) [64]. A full comparison, including detector material, electrode configuration, and measurement conditions, is reported in Table S1. The dynamic response further confirms this behavior: during irradiation, the stacked device shows a strong, steady signal, while the planar one exhibits a much lower response (Figure 3C). When comparing the proton‐induced currents measured at a fixed flux (3 × 10^9^ H^+^ cm^−^^2^ s^−^^1^) at different electric fields (Figure 3D), the advantage of the stacked configuration becomes evident. While planar devices show only modest improvements with increasing field, the stacked ones exhibit a stronger field dependence, with significantly higher output and enhanced charge collection as the field increases. This different trend is linked to the geometry and electric field distribution, i.e., the stacked structure enables more effective collection throughout the entire volume.

**FIGURE 4:**
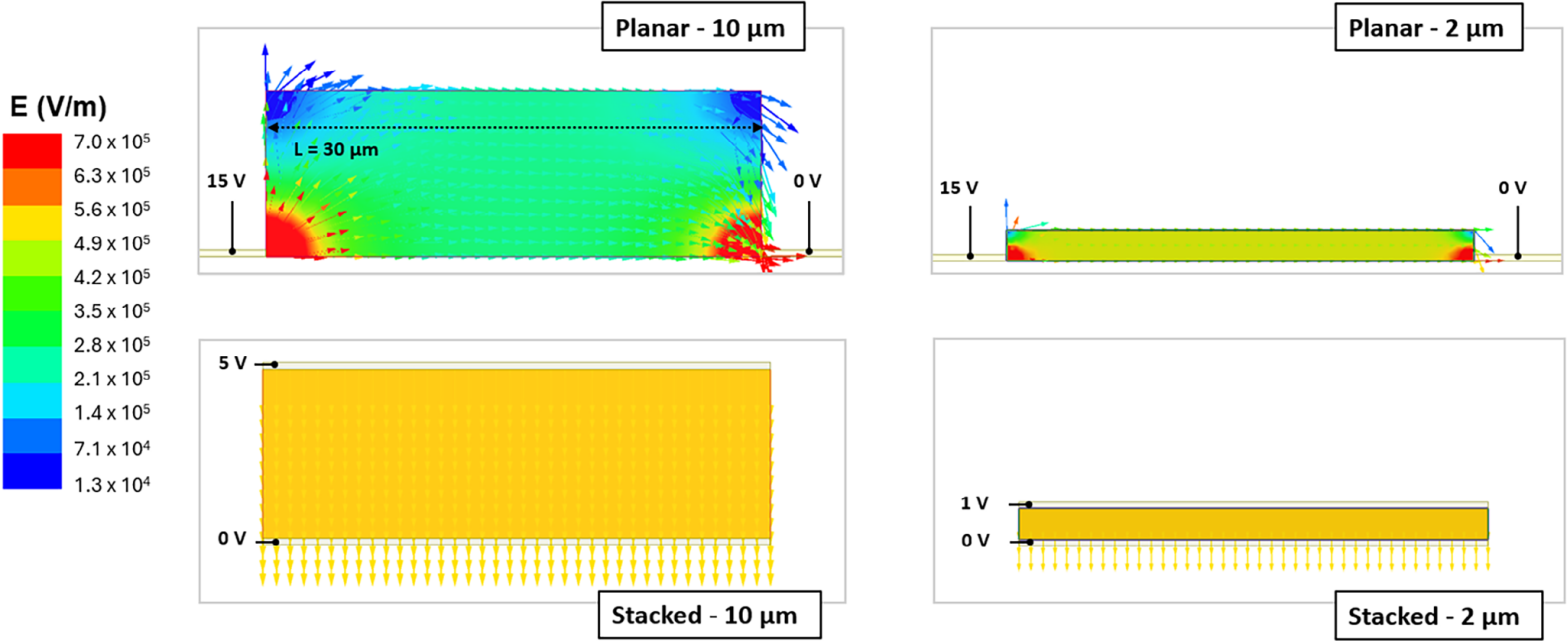
Electric field simulations were carried out using the ANSYS Maxwell 2D software, considering both planar (top) and stacked (bottom) architectures. For planar ones, the geometry was built to reproduce the real structure of the semiconducting channel (30 µm) between a single pair of fingers of the interdigitated electrodes. For the vertical ones, the distance between the electrodes corresponds to the thickness of the perovskite layer. The simulations were performed for both the two thicknesses, 2 and 10 µm. The electrodes were modeled with 40 nm of gold. The perovskite was assigned an electrical conductivity of 1×10^−^^1^ ^1^ S m^−1^ and a relative permittivity ε_r_ = 3. As for the applied voltages, 15 V were used in the planar configuration, while 1 and 5 V were applied to the 2 and 10 µm vertical configurations, respectively. These values were chosen to establish an ideal electric field of approximately 0.5 V µm^−1^ across the active layer.

This effect is supported by ANSYS Maxwell 2D simulations (Figure 4), which show the electric field distribution in both planar (top) and stacked (bottom) geometries for perovskite layers with 2 µm (right) and 10 µm (left) thicknesses. In the planar configuration, the electrodes are separated by a 30 µm gap, while in the stacked devices the distance between electrodes corresponds to the perovskite thickness. The material was modeled with an electrical conductivity of 1 × 10^−^^1^ ^1^ S m^−1^ and a relative permittivity ε_r_ = 3 [65, 66, 67]. Voltage values were chosen to generate an ideal field of 0.5 V µm^−1^ across the active layer, resulting in applied biases of 15 V for the planar case, and 1 and 5 V for the 2 and 10 µm stacked configurations, respectively. As shown in the simulations, the planar geometry presents a strong field concentration near the electrodes, with peak values exceeding 0.5 V µm^−1^ (as indicated in red on the color scale). However, the field rapidly drops just a few microns into the bulk, below 0.3 V µm^−1^in the 10 µm thick case, resulting in poor field penetration. This creates a highly non‐uniform distribution, with an effective average field of only ∼0.26 V µm^−^^1^—approximately half of the nominal 0.5 V µm^−^^1^. Even in the 2 µm‐thick planar configuration, the field is significantly less homogeneous: while local maxima occur near the electrodes, the average field across the active volume is about 0.46 V µm^−1^. On the other hand, the stacked architecture shows a much more uniform vertical field, with intensity equal to the target value of 0.5 V µm^−1^ (yellow in the color scale) throughout the entire layer, both for 2 and 10 µm thicknesses. The field lines remain straight and dense across the full depth, enabling efficient charge collection and ensuring shorter and more direct carrier transport paths, reducing the chance of recombination. A full quantitative comparison of the simulated average fields at all nominal applied fields is reported in Table S2, confirming that planar devices systematically operate under lower effective fields than their stacked counterparts. This difference in electric field distribution and homogeneity leads to a difference in the volume of the active layer actually involved in transport: while in the stacked architecture, all regions of the film experience essentially the same vertical field and therefore contribute uniformly to charge collection, the planar device operates with regions subject to markedly different field strengths, resulting in portions of the film that collect charges efficiently and others where the collection is significantly less effective. As a consequence, the two architectures do not exploit the same effective active volume, and this mismatch directly results in the higher sensitivity measured in the stacked configuration.

### 2.2 Proton Detection by 10 µm PEA_2_PbBr_4_ Film Stacked Architecture

**FIGURE 5:**
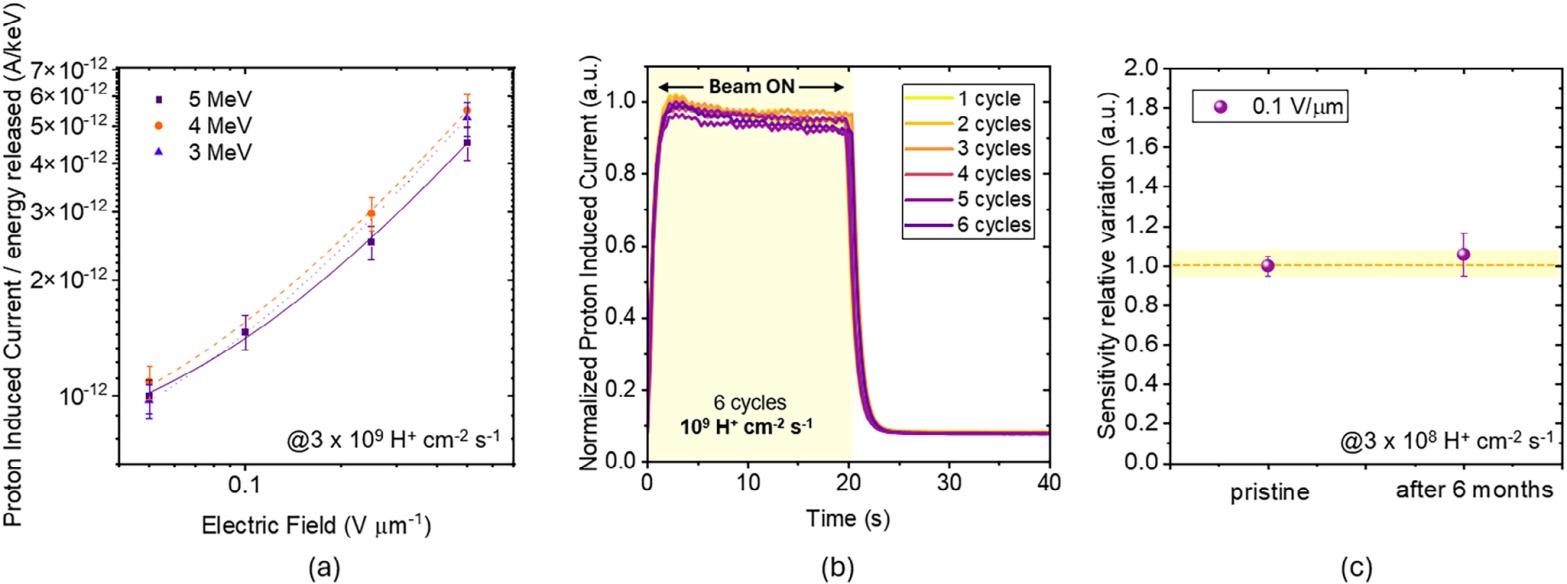
(a) Dependence of proton‐induced current, normalized to the released energy, on the applied electric field for protons with beam energies of 3 MeV (blue), 4 MeV (orange), and 5 MeV (purple), at 3 × 10^9^ H^+^s^−1^cm^−2^. (b) Reproducibility test. Plot of the dynamic current response as a function of time of the detector biased at 0.05 V µm^−1^and irradiated with 20 s proton beam shots. The irradiation time window is indicated as a yellow region. Six irradiations were performed at a flux (1.3 ± 0.2) × 10^9^ H^+^s^−1^cm^−2^. (c) Aging test. Comparison between the performance of the detector, with an applied electric field of 0.1 V µm^−1^, in pristine condition and after 6 months. The reported performance refers to the sensitivity relative variation, extracted at 3 × 10^8^ H^+^ cm^−2^ s^−1^. The yellow band represents the ±5% range around the initial sensitivity; the value remains within this range, indicating robust long‐term operational stability.

The thickest stacked device (10 µm) was selected for a detailed characterization to test its primary beam energy dependence and long‐term operational stability under irradiation. Specifically, it was chosen as it maximizes the energy deposition from incoming protons. To evaluate whether the detector response depends on the energy of the incoming protons, the same device was irradiated with protons of 3, 4, and 5 MeV energy at 3 × 10^9^ H^+^ s^−1^ cm^−2^ proton flux. The values of energy released reported in Table 1 highlight that even a small change in the primary beam energy leads to a significant variation in the released energy. Figure 5A shows the resulting curves of the induced current as a function of the electric field, ranging between 0.05 and 0.5 V µm^−1^, normalized by the released energy. The responses corresponding to the three energies are comparable, within 10% error, over the entire range of applied fields, pointing out the detector's beam energy‐independent response in the investigated energy window. Such behavior indicates that the charge collection and transport through the active layer are not significantly affected by the variation of proton energy beam in this range and confirms the stable performance of the detector's operation under varying irradiation conditions. Moreover, the detector's Limit of Detection (LoD) was evaluated, as shown in Figure S9, by measuring the device response under proton irradiation up to a flux of 2.5 × 10^6^ H^+^ cm^−^^2^ s^−^^1^ at 3 MeV, with an applied bias of 5 V. Figure S5 also reports the corresponding signal‐to‐noise ratio (SNR), calculated using the RMS value of the dark signal (0.144 ± 0.016) pA; under these conditions, a flux of 2.5 × 10^6^ H^+^ cm^−^^2^ s^−^^1^ yields an SNR of 10^2^. This value is approximately four orders of magnitude lower than that reported for a MAPbBr_3_ single crystal irradiated with a 3 MeV proton beam, which exhibited a LoD of about 2.07 × 10^1^ ^0^ H^+^s^−^cm^−^^2^ under an electric field of 0.01 V µm^−1^ [64]. The reliability of this material system was already demonstrated in our previous studies. Devices employing this 2D perovskite have shown stable X‐ray photoconductive response up to cumulative doses of 9.6 Gy [31], with no measurable degradation in sensitivity, and an essentially unchanged dynamic response even after 80 days of aging following exposure to 3.4 Gy, biased at 80 V [49]. In addition, electrical characterization of PEA_2_PbBr_4_ devices has revealed highly robust environmental stability: the current–voltage curves measured immediately after fabrication and again after four months of storage in air, at room temperature and in the dark, are identical, indicating no drift of the transport properties over time [33]. In this work, we also directly evaluated both the reproducibility under repeated irradiation cycles and the long‐term stability of the stacked detector's response.

As shown in Figure 5B, the sensor was exposed to six consecutive proton pulses at a flux of (1.3 ± 0.2) × 10^9^ H^+^ s^−^^1^ cm^−^^2^, for a total of 6 × 10^9^ H^+^, corresponding to 176 Gy. The current signals recorded for each cycle are almost perfectly overlapping, with no visible degradation nor variation in peak amplitude/signal shape across the six exposures. This excellent reproducibility indicates a stable detection response under subsequent irradiation cycles up to a total irradiation of 1.6 × 10^11^ H^+^ cm^−2^ and confirms the absence of transient or cumulative effects over the tested fluence range. In addition, the device's long‐term operational stability was evaluated by repeating the detector response characterization under proton irradiation after six months of storage in ambient conditions (in dark, in air, and without encapsulation, with indoor temperature ranging from approximately 18°C–25°C and relative humidity fluctuating between roughly 40%–60%). Figure 5C shows the variation of the sensitivity, acquired at 3 × 10^8^ H^+^ s^−^^1^ cm^−^^2^, applying an electric field of 0.1 V µm^−1^, relative to its pristine value. The comparison reveals no statistically significant variation between the pristine and aged device. The response remains within a narrow band (highlighted yellow), with a fluctuation of 5%. This result is fully aligned with the known intrinsic stability of 2D perovskites: layered architecture and large organic spacer cations significantly contribute to increase hydrophobicity and strongly suppress ion migration, which represent two of the main degradation pathways in 3D perovskites [25]. Therefore, it highlights the potential of the device for long‐term applications without the need for encapsulation or controlled storage conditions.

## 3 Conclusion

This study highlights the critical role of device architecture in controlling the performance of 2D perovskite‐based proton detectors. While the capability of these materials to detect protons was already established, our results show that switching from a planar to a stacked geometry leads to a significant enhancement in charge collection efficiency and overall detecting signal output. The stacked geometry enables a more effective exploitation of the active volume, allowing charges generated throughout the perovskite layer to be efficiently collected, even at low bias. This advantage is particularly evident in thinner devices (with an active layer approximately 2 µm thick), which still exceed by one order of magnitude the thicknesses typically adopted in previous works, where morphological uniformity further supports charge transport. In contrast, the planar layout is limited by its lateral field configuration and in‐plane‐constrained collection. Our results also demonstrate that stacked devices show excellent stability and reproducibility over time, with no degradation observed after prolonged storage. Additionally, the detector response shows remarkable energy independence within the 3–5 MeV proton range, confirming the robustness of the charge generation and transport mechanisms across varying LET conditions. Although thicker films (up to 10 µm) promise higher sensitivity, their performance remains limited by morphological issues that limit charge mobility, highlighting the need for further optimization in thicker layers. In summary, this study demonstrates that the architecture of perovskite‐based detectors is a key design parameter to control the resulting detectors performance. The stacked configuration represents a highly effective strategy to unlock the full potential of these materials for radiation detection applications, especially where compact, flexible, and low‐power devices are required.

## 4 Methods

### 4.1 Device Fabrication

Both devices were fabricated on 75 µm thick polyimide substrate. The substrates were cleaned by subsequent ultrasonic baths in acetone, isopropyl alcohol, and deionized H_2_O, for 5 min each. For planar electrodes lithographic techniques were employed to design the interdigitated pattern. A positive photoresist (S1818) was spin coated on the substrate at 4000 rpm for 60 s. The layout was designed by CleWin software and patterned using an optical Microwriter (ML3, Durham Magneto Optic) to expose the resist. The development was carried out using MICROPOSIT MF‐139 developer, followed by rinsing in deionized water. The electrodes, consisting of a 6 nm chromium adhesion layer and a 35 nm gold layer, were deposited by thermal evaporation. Patterning was achieved by rinsing the entire structure in an acetone bath for 4 h (lift‐off process). Vertical electrodes were instead patterned using a physical mask during the evaporation. The same electrode composition (6 nm Cr/ 35 nm Au) was used for this structure. In both planar and vertical configurations, the electrode area was 2 × 2 mm^2^.

For the perovskite active layer, a 0.7 m solution was prepared by mixing C_6_H_5_C_2_H_4_NH_3_Br (PEABr, Sigma–Aldrich > 98%) and PbBr_2_ (Sigma–Aldrich > 98%) in N,N‐dimethylformamide (DMF, Sigma–Aldrich 99.8% anhydrous). Everything was performed inside a nitrogen‐filled glovebox. The solution was mixed thoroughly for 5 h until complete dissolution of the precursors and filtered.

The perovskite layer was deposited by spin coating (800 rpm 60s; 800 rpm 5s + 6000 rpm 55s) and annealed for 10 min of annealing at 50°C. During spin coating, chlorobenzene was used as an antisolvent and dropped onto the substrate shortly after dispensing the perovskite solution.

To complete the fabrication of the vertical samples, a gold electrode was thermally evaporated on top of the perovskite layer.

### 4.2 Profilometer Characterization

Film thickness and surface roughness were evaluated by acquiring images of the surface using the GBS smartWLI white light interferometer with a 10x magnification lens. Specifically, the first was estimated by extracting a line profile across the edge between the perovskite layer and the Kapton substrate. The latter, by selecting a region of the thin film surface.

The subsequent data analysis was carried out with Mountains 9 software. Different regions from four samples of each type were analyzed to obtain the reported values, and the associated error was calculated based on the variation in the mean among these measurements.

### 4.3 X‐ray Diffraction

XRD measurements were performed using a Malvern Panalytical Empyrean diffractometer operating in Bragg–Brentano geometry (reflection mode) over a 2θ range of 3°–50°. The instrument was equipped with a scintillation detector and a monochromator. Data were collected using a θ–2θ scan method with Cu Kα radiation (weighted average wavelength = 1.5406 Å; Kα_1_ = 1.5406 Å, Kα_2_ = 1.5445 Å, intensity ratio Kα_1_:Kα_2_ = 2:1).

### 4.4 Scanning Electron Microscope

The images were acquired by using a Cambridge Stereoscan 360 SEM, operating at 20 kV. The cross‐section samples were obtained by cutting the Kapton substrate at the edge and pulling apart two pieces by hand.

### 4.5 Atomic Force Microscopy

AFM measurements were performed in non‐contact mode by using the Park NX‐10. The cantilever was the NSC36 (MikroMasch) made of n‐type silicon and coated with a layer of chromium and gold (Force Constant ∼2 N/m; Resonance Frequency of ∼70 kHz).

### 4.6 Electrical Characterization

Electrical characterization was carried out under dark conditions using a probe station equipped with a commercial electrometer. Current‐voltage (IV) curves were acquired for both planar and stacked architecture. For planar devices, the sheet resistance was calculated as $R_{S}=R\frac{W}{L}\;$, where *W* is the channel width (50 mm), and *L* is equal to the channel length (30 µm). For stacked devices, the current path was assumed to be vertical, and the resistivity was estimated as $\rho =R\frac{L}{A}\;$, *A* was taken as the electrode area, while *L* corresponded to the thickness (2 or 10 µm, respectively).

### 4.7 Proton Irradiation

The characterization of the perovskite thin film detectors was performed at the LABEC ion beam facility (INFN Florence, Italy). The 3, 4, 5 MeV proton beam delivered by the 3 MV Tandetron accelerator was employed for the present study. The proton beam was extracted into ambient atmosphere through a 200 nm thick Si_3_N_4_ membrane, with the sample positioned at 8 mm from the extraction window. Specifically, the samples were closed in a metal box to prevent the illumination of the device during the irradiation and to reduce the electrical noise. A small aperture was present to let the proton beam in (covered by a 14 µm Al foil). During the experiments, beam currents ranged from 10^−1^ to 10^2^ pA. Owing to the low intensity of the beam, its current was continuously monitored and quantitatively assessed using a rotating chopper placed between the beam extraction membrane and the sample. The chopper, consisting of a graphite vane coated with a thin layer of nickel, enables indirect measurement of the proton flux through the detection of Ni Kα X‐ray emission.

To accurately determine the energy of the protons reaching the active layer of the detector, the energy loss due to the materials interposed between the extraction point and the sensor was calculated. These include: a 200 nm thick Si_3_N_4_ membrane used for beam extraction, an 8 mm gap filled with a 50/50 air–helium mixture between the membrane and the enclosure, a 14 µm thick aluminum entrance window of the metal box housing the sensor, and an additional 14 mm of air inside the box. The total energy loss associated with these layers amounts to approximately 390 keV, as estimated using SRIM Monte Carlo simulations.

Samples were irradiated with proton flux in the range [10^6^–10^10^] H^+^ cm^−2^ s^−1^. The spot of the proton beam has an area of 0.17 cm^2^.

The electrical signal was acquired by a commercial electrometer.

## Conflicts of Interest

The authors declare no conflicts of interest.

## Supporting information




**Supporting File**: smll72379‐sup‐0001‐SuppMat.docx.

## Data Availability

The data that support the findings of this study are available from the corresponding author upon reasonable request.
