# Supplementary material for: Impact of Device Architecture on Proton Detection Efficiency in 2D Perovskite Thick Film Detectors
Source: Small. 2026 Jan 25;22(15):e12236. doi: 10.1002/smll.202512236 (PMC12980477; doi:10.1002/smll.202512236)
Supplement: Supplementary file 1 — Supporting File: smll72379‐sup‐0001‐SuppMat.docx. [file SMLL-22-e12236-s001.docx]

Supporting Information

Impact of Device Architecture on Proton Detection Efficiency in 2D Perovskite Thick Film detectors

Giulia Napolitano, S. Cepić, I. Fratelli*, M. Chiari, B. Fraboni and L. Basiricò

**
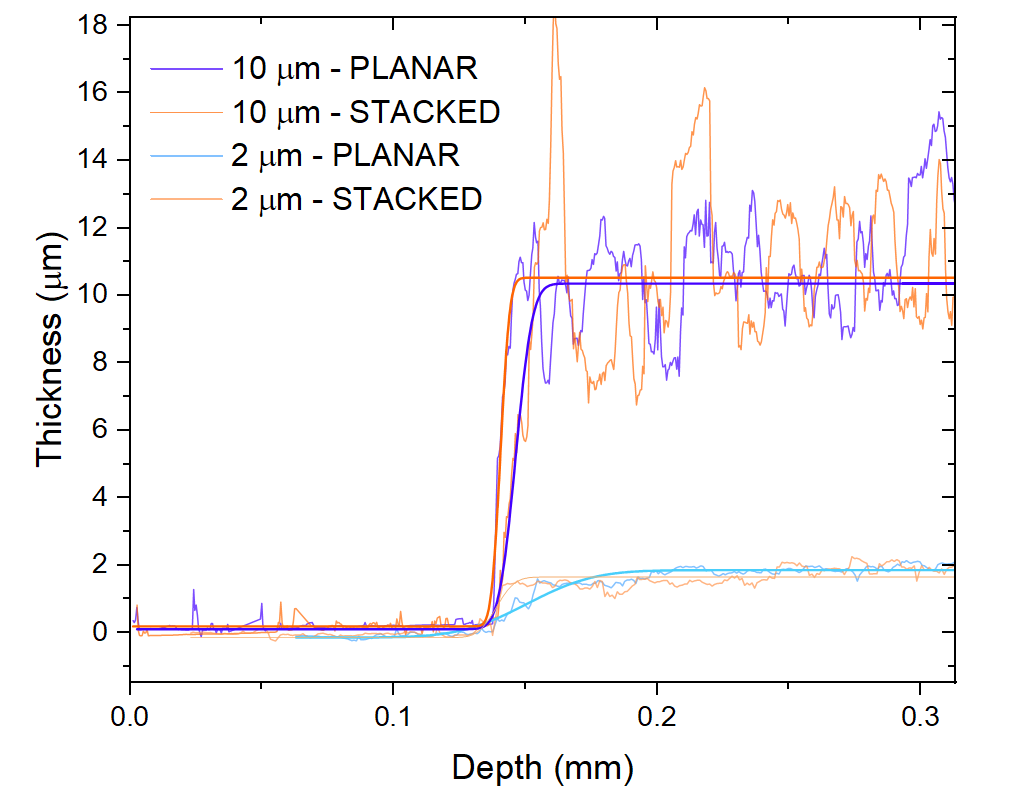
**

**Figure S1. Thickness of perovskite thin films deposited at different speeds.** Profiles of the perovskite layers deposited at different spin coating speed, for both planar and stacked architectures.


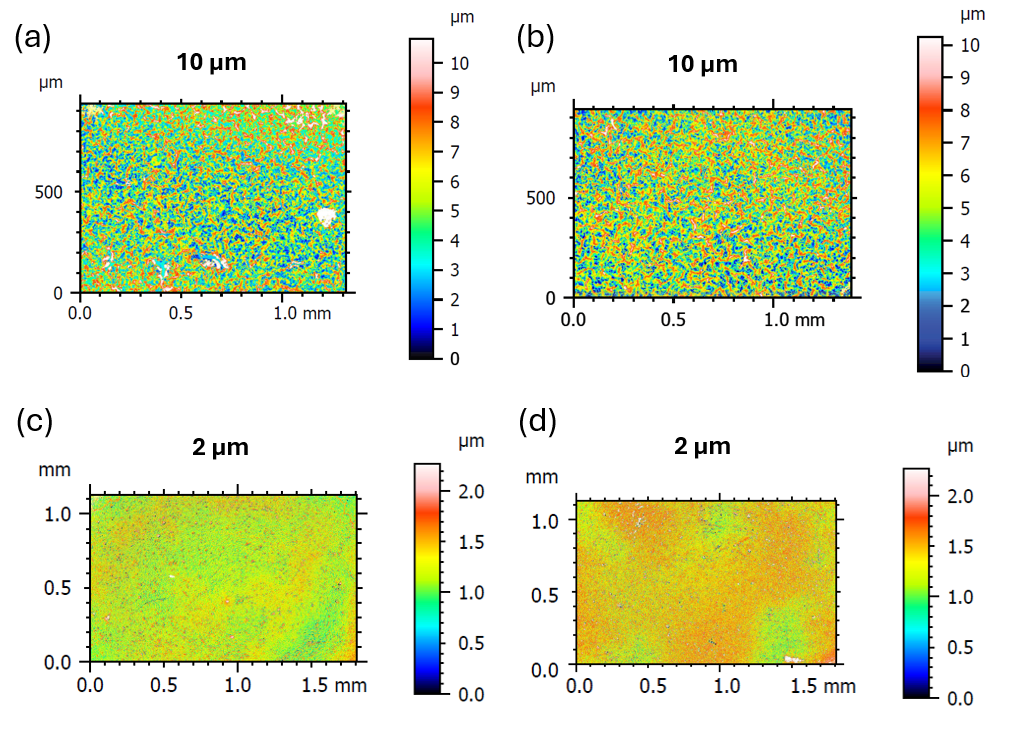


**Figure S2.** Optical profilometry maps of PEA₂PbBr₄ films with thicknesses of 10 µm ((a), (b)) and 2 µm ((c), (d)), acquired at two regions located at opposite edges of the 2 cm² substrate.

**
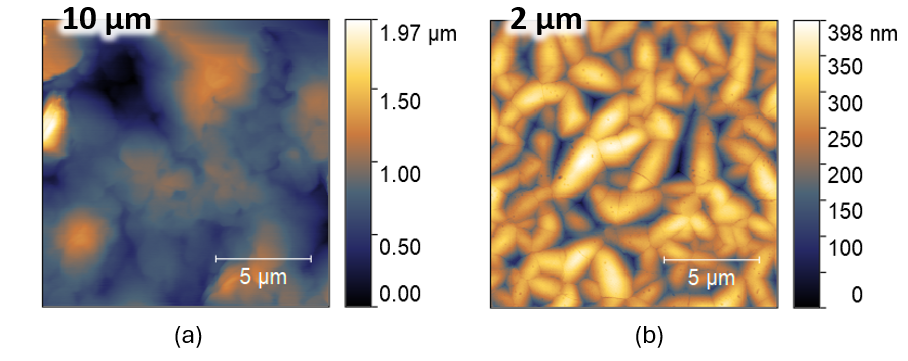
**

**Figure S3.** AFM height maps of PEA₂PbBr₄ thin films: (a) 10 µm-thick and (b) 2 µm-thick films.

**
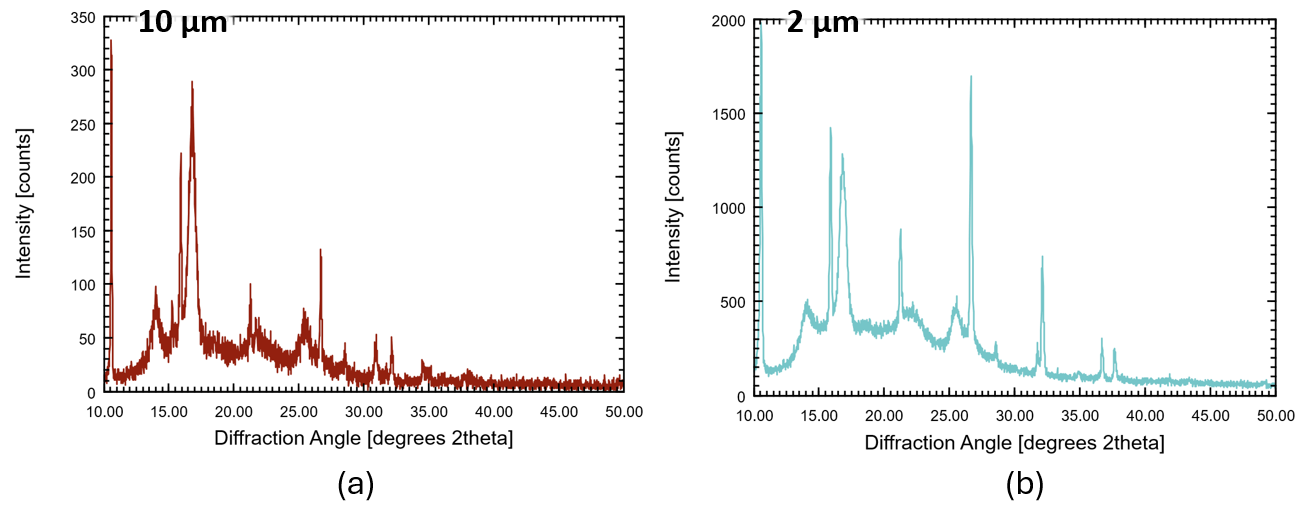
**

**Figure S4.** XRD of (a) 10 and (b) 2 µm PEA₂PbBr₄ films. The diffractograms show that all samples exhibit reflections characteristic of the layered perovskite phase PEA₂PbBr₄, with major peaks at 2θ = 10.64°, 15.88°, 21.25°, 26.73°, 32.23°, and 37.70° corresponding to the (001)–(007) planes, respectively. Additional reflections originating from the Kapton substrate are observed at 2θ = 14.01°, 16.83°, 18.58°, and 25.53°.


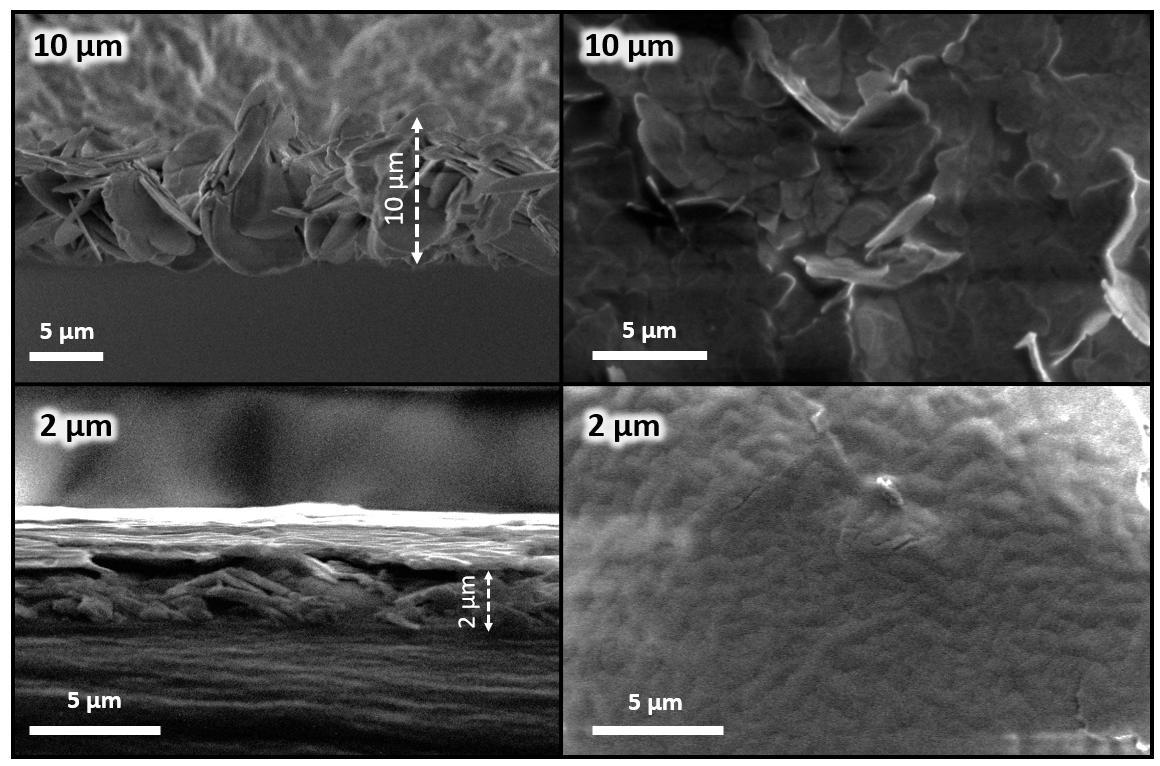


**Figure S5. PEA_2_PbBr_4_ microstructure for different thicknesses.** Top-view (right) and cross-section (left) SEM images of polycrystalline PEA₂PbBr₄ thin films with thicknesses of 10 µm (top row) and 2 µm (bottom row).

**
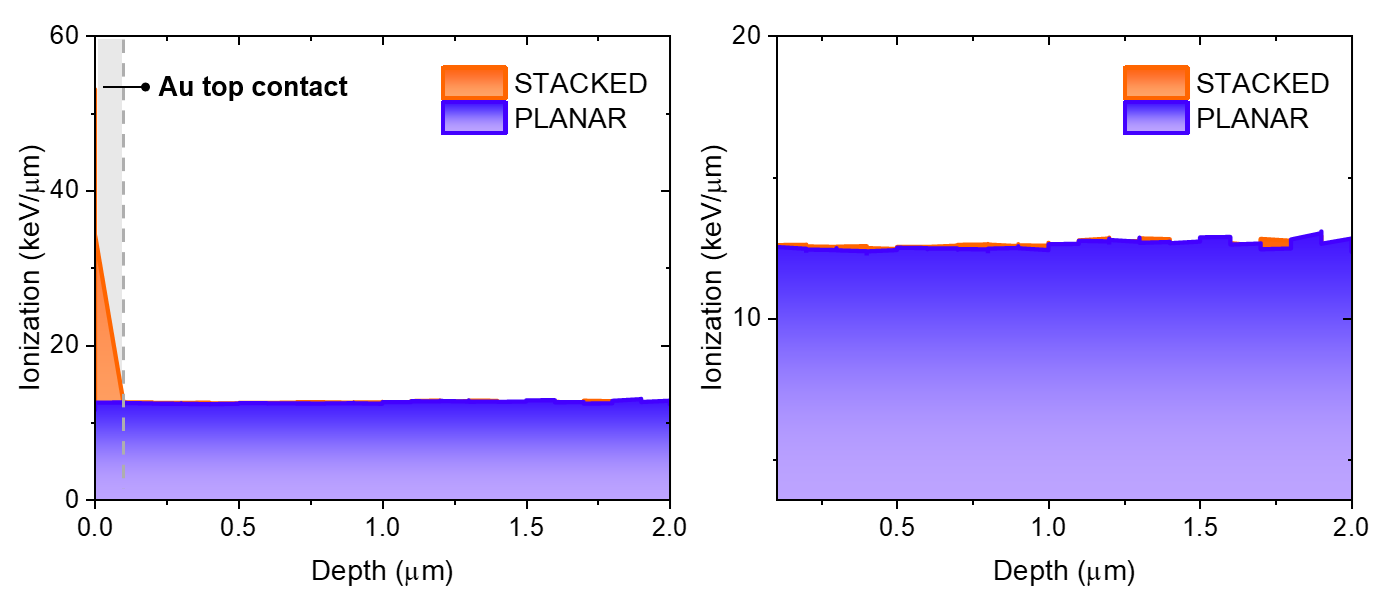
**

1. ***2 µm, 5 MeV***

**
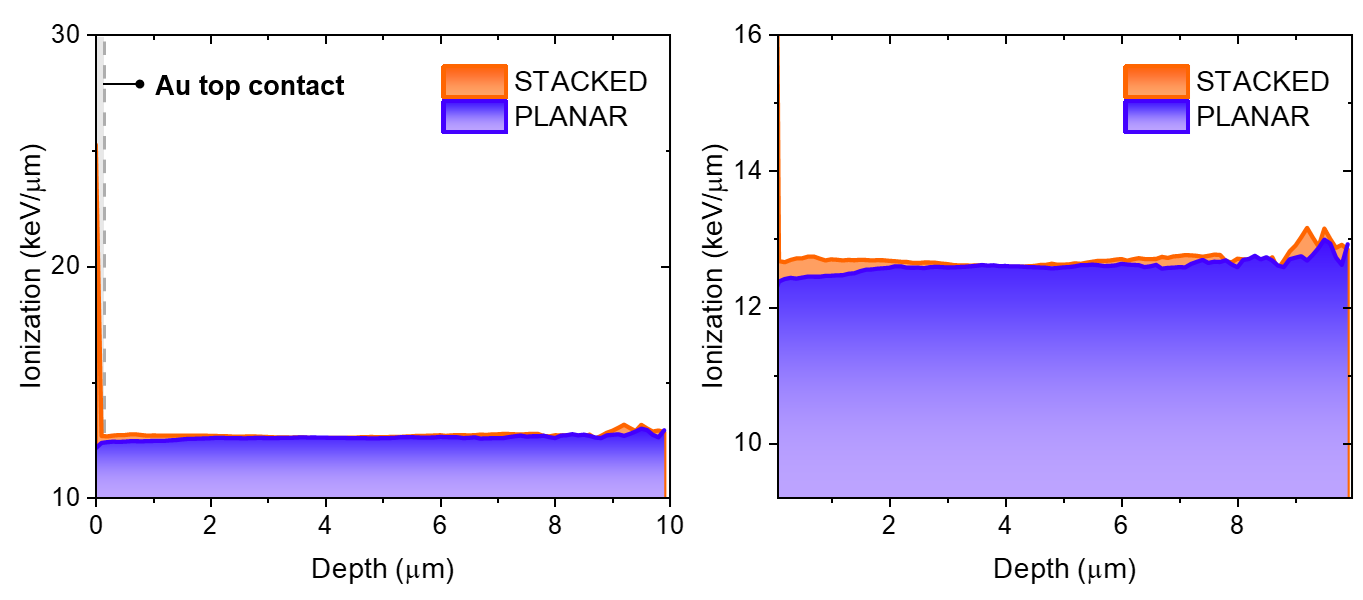
**

1. ***10 µm, 5 MeV***

**Figure S6. SRIM simulation.** Simulated ionization profiles for planar and stacked perovskite detectors under proton irradiation. (a, b) LET distributions for 5 MeV protons in 2 μm and 10 μm thick layers, respectively; the stacked configuration includes a 40 nm Au top contact (grey region). The zoomed-in views highlight the overlap between the two simulations in the two architectures.


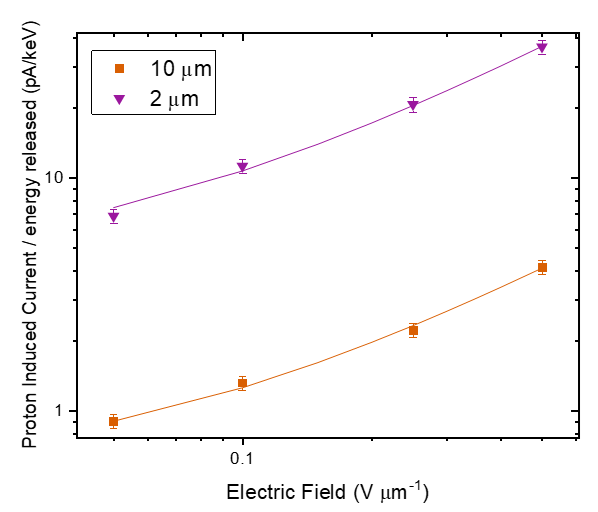


**Figure S7.** (a) Comparison of proton-induced current at 3 x ${10}^{9} H^{+}{s^{-1}\mathrm{cm}}^{-2}$, normalized by the energy released, for the stacked detectors with different thicknesses: 10 µm (purple) and 2 µm (orange), under varying electric fields from 0.05 to 0.5 V/µm.


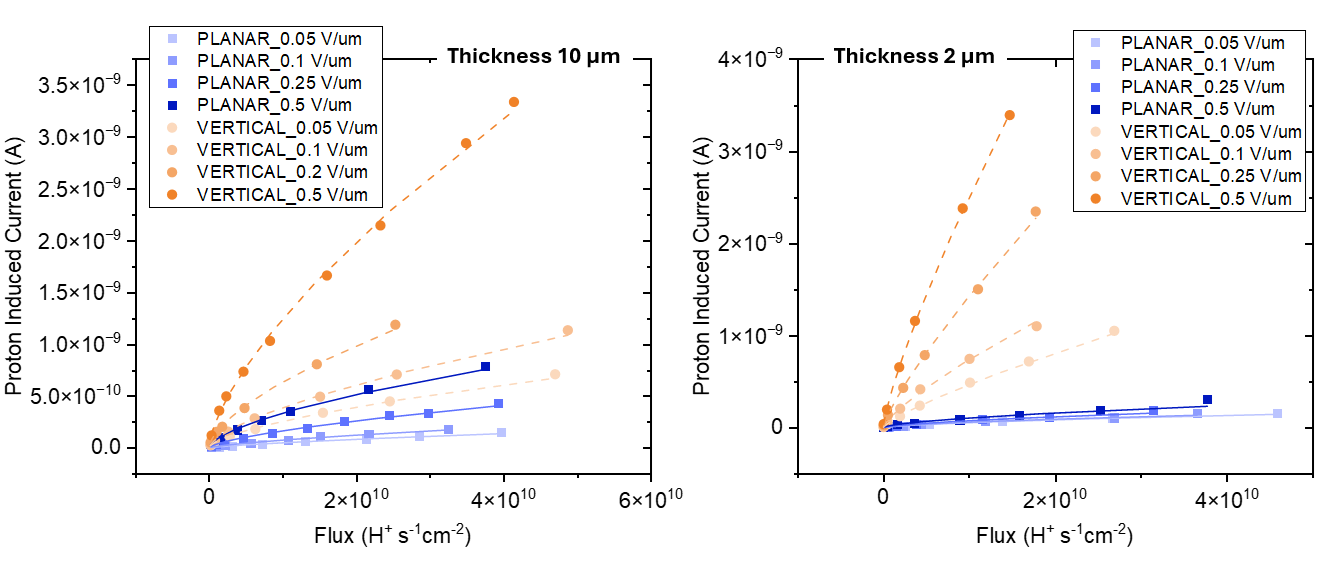


**Figure S8. Proton induced current vs. proton flux in linear scale.** Both vertical (orange) and planar (blue) architectures were tested under proton fluxes between 10^8^ – 10^10^ H^+^ s^-1^ cm^-2^ . The applied electric field was in the range between 0.05 and 0.5 V µm^-1^.

| MATERIAL | Architecture | Electric Field (V µm^-1^) | Proton Energy (MeV) | Sensitivity (C H^+-1^) | | REF. |
| --- | --- | --- | --- | --- | --- | --- |
| PEA_2_PbBr_4_ | Stacked (Au/ PEA_2_PbBr_4_/Au) | 0.5 | 5 | | (1.0 ± 0.2)10^-17^ | This work |
| MAPbBr_3_ | Stacked (Ag/ PEA_2_PbBr_4_/Ti/Au) | 0.01 | 3 | | (2.19 ± 0.03)10^-18^ | **[64]** |
| CsPbCl_3_ | Co-planar Pd electrodes | 2 | 100-228 | | 4·10^-20^ | **[34]** |
| TIPGe-Pentacene | Co-planar Au electrodes | 0.03 | 5 | | (6.4 ± 0.2)10^-20^ | **[63]** |
| MAPbBr_3_+  PEA_2_PbBr_4_ | Co-planar Au electrodes | 0.2 | 5 | | (1.12 ± 0.01)10^-18^ | **[51]** |

**Table S1.** **Sensitivity for different proton detectors.** Overview of proton-detection sensitivities from previous studies using various active-layer materials and device architectures.


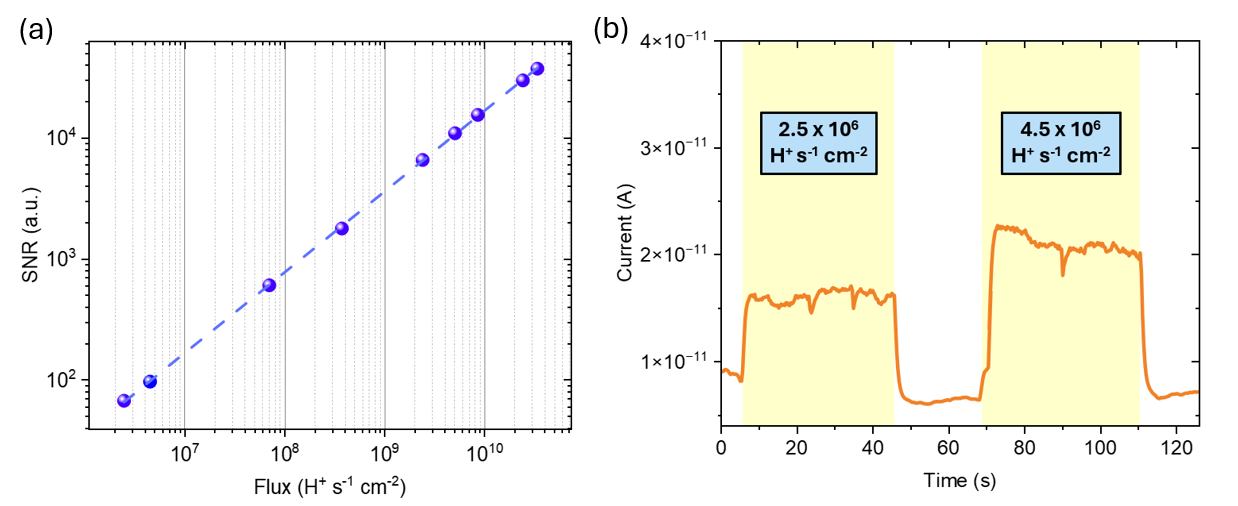


**Figure S9. LoD for 10 µm PVK thin film stacked device.** (a) Plot of the SNR as function of the proton flux. The 10 µm stacked device was irradiated at a proton energy beam of 3 MeV and biased at 5 V. (b) Dynamic plot of current vs. time at the last two proton fluxes (4.5× 10^6^ H^+^ s^-1^ cm^-2^ and 2.5 × 10^6^ H^+^ s^-1^ cm^-2^).

| Nominal Electric Field (V µm^-1^) | Average Simulated Electric Field (V µm^-1^) | | | |
| --- | --- | --- | --- | --- |
|  | PLANAR 10 µm (L=30 µm) | PLANAR 2 µm (L=30 µm) | STACKED 10 µm (L=10 µm) | STACKED 2 µm (L=2 µm) |
| 0.5 | 0.26 | 0.46 | 0.5 | 0.5 |
| 0.25 | 0.13 | 0.23 | 0.25 | 0.25 |
| 0.1 | 0.053 | 0.093 | 0.1 | 0.1 |
| 0.05 | 0.026 | 0.046 | 0.05 | 0.05 |

**Table S2.** **Electric field simulation.** Nominal electric field and simulated average electric field across the semiconductor layer for all investigated film thicknesses and electrode geometries. The first is calculated as the ratio between the applied bias and the channel length, while the second is extracted from Ansys simulations accounting for the actual field distribution arising in each architecture.
